# Supplementary material for: Essential Oils Distilled from Colombian Aromatic Plants and Their Constituents as Penetration Enhancers for Transdermal Drug Delivery
Source: Molecules. 2023 Mar 22;28(6):2872. doi: 10.3390/molecules28062872 (PMC10057770; doi:10.3390/molecules28062872)
Supplement: Supplementary file 1 [file molecules-28-02872-s001.zip › Figures S1_S2_P_Escobar_Molecules (1) (1).pptx]

## Slide 1
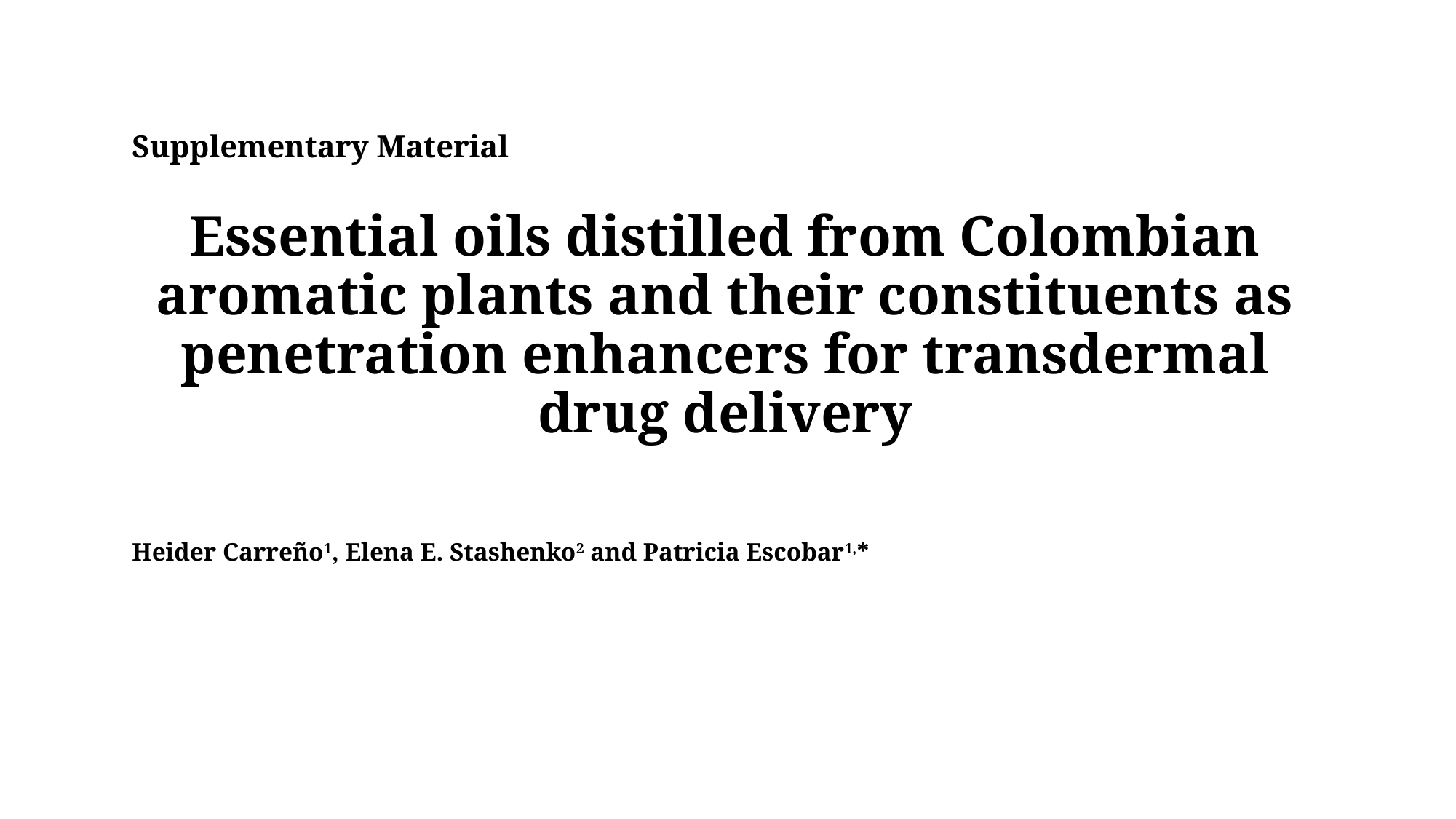

Supplementary Material
Essential oils distilled from Colombian aromatic plants and their constituents as penetration enhancers for transdermal drug delivery
Heider Carreño1, Elena E. Stashenko2 and Patricia Escobar1,*

## Slide 2
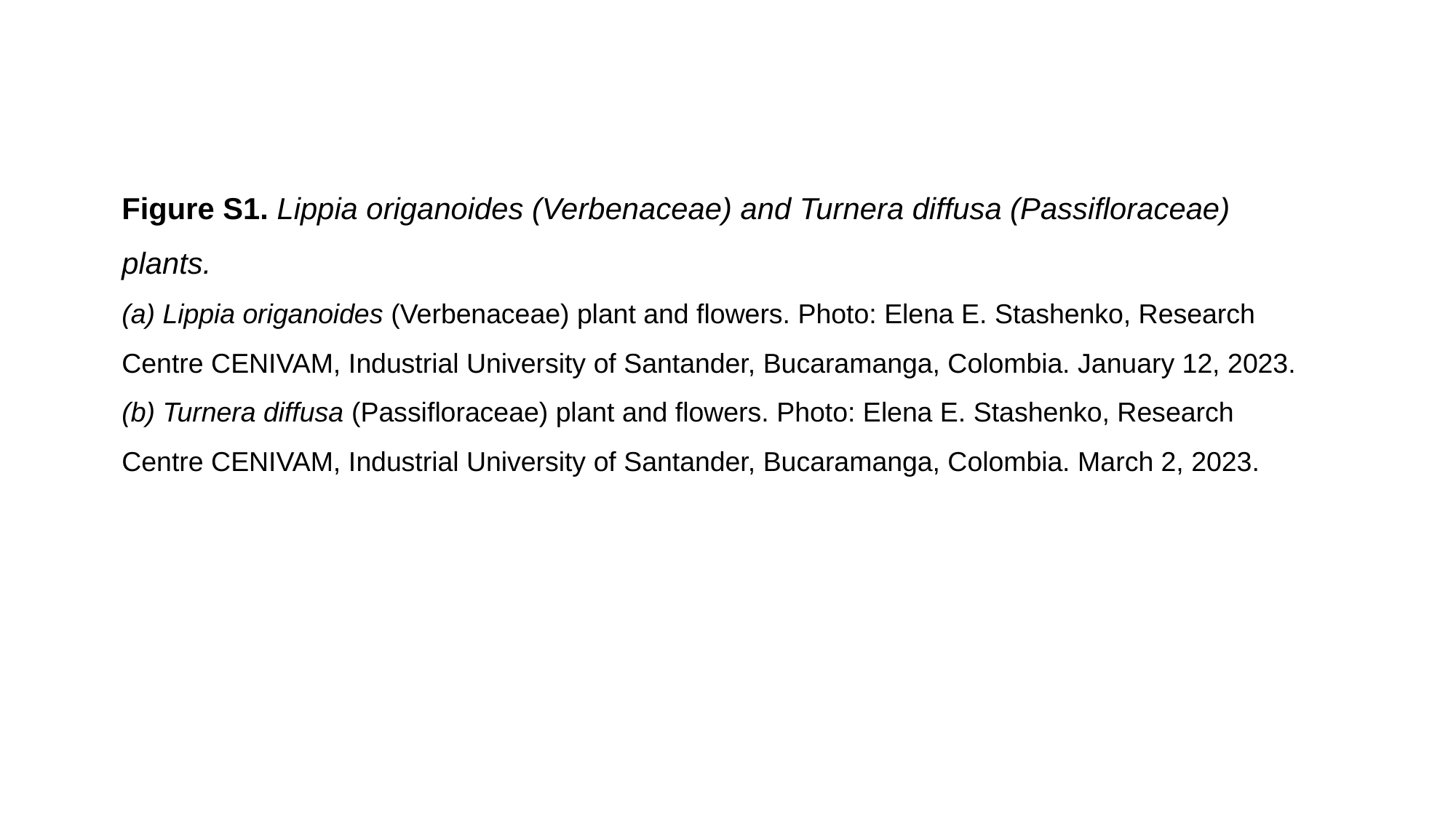

Figure S1. Lippia origanoides (Verbenaceae) and Turnera diffusa (Passifloraceae) plants.
(a) Lippia origanoides (Verbenaceae) plant and flowers. Photo: Elena E. Stashenko, Research Centre CENIVAM, Industrial University of Santander, Bucaramanga, Colombia. January 12, 2023.
(b) Turnera diffusa (Passifloraceae) plant and flowers. Photo: Elena E. Stashenko, Research Centre CENIVAM, Industrial University of Santander, Bucaramanga, Colombia. March 2, 2023.

## Slide 3
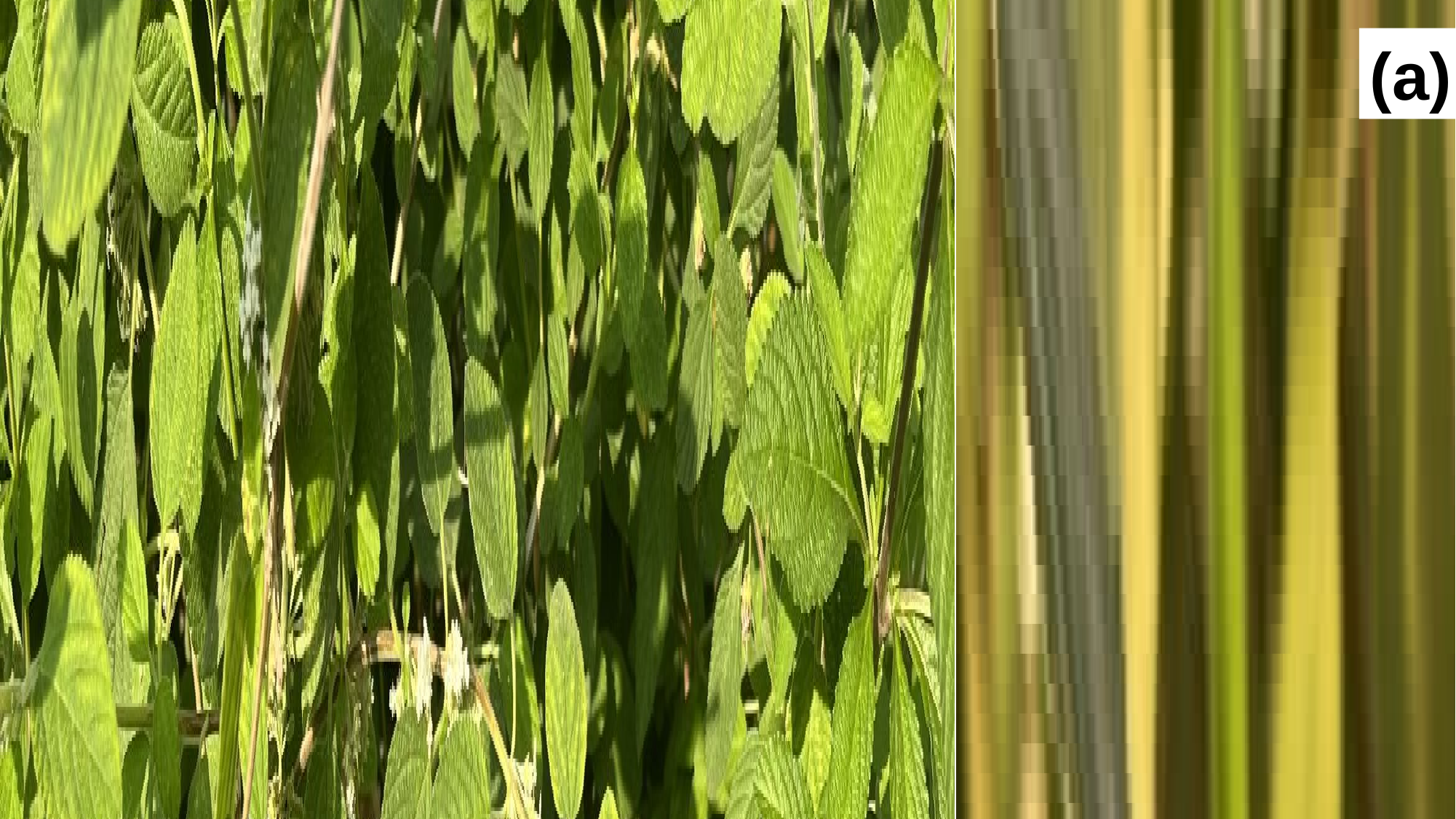

(a)

## Slide 4
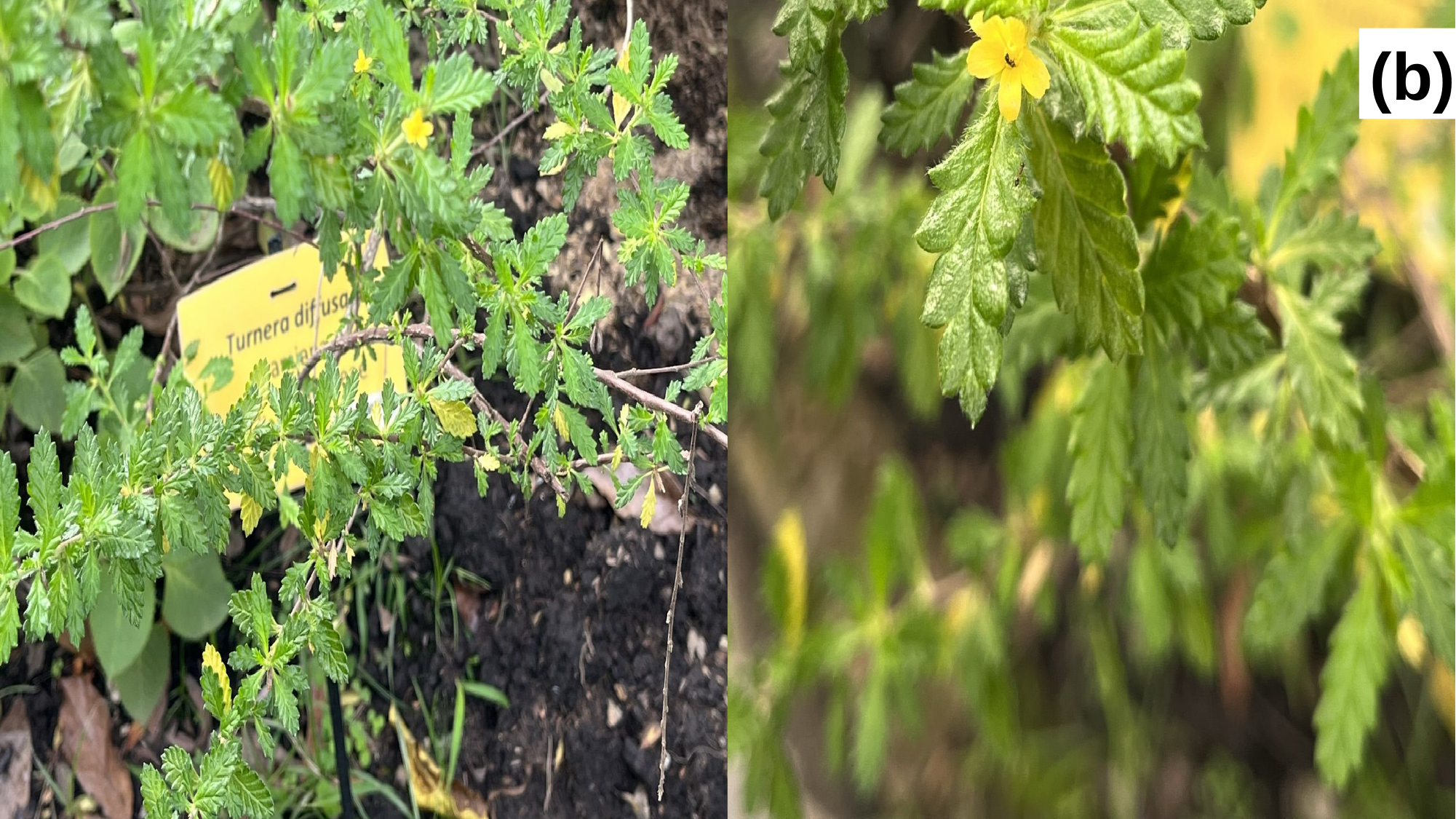

(b)

## Slide 5
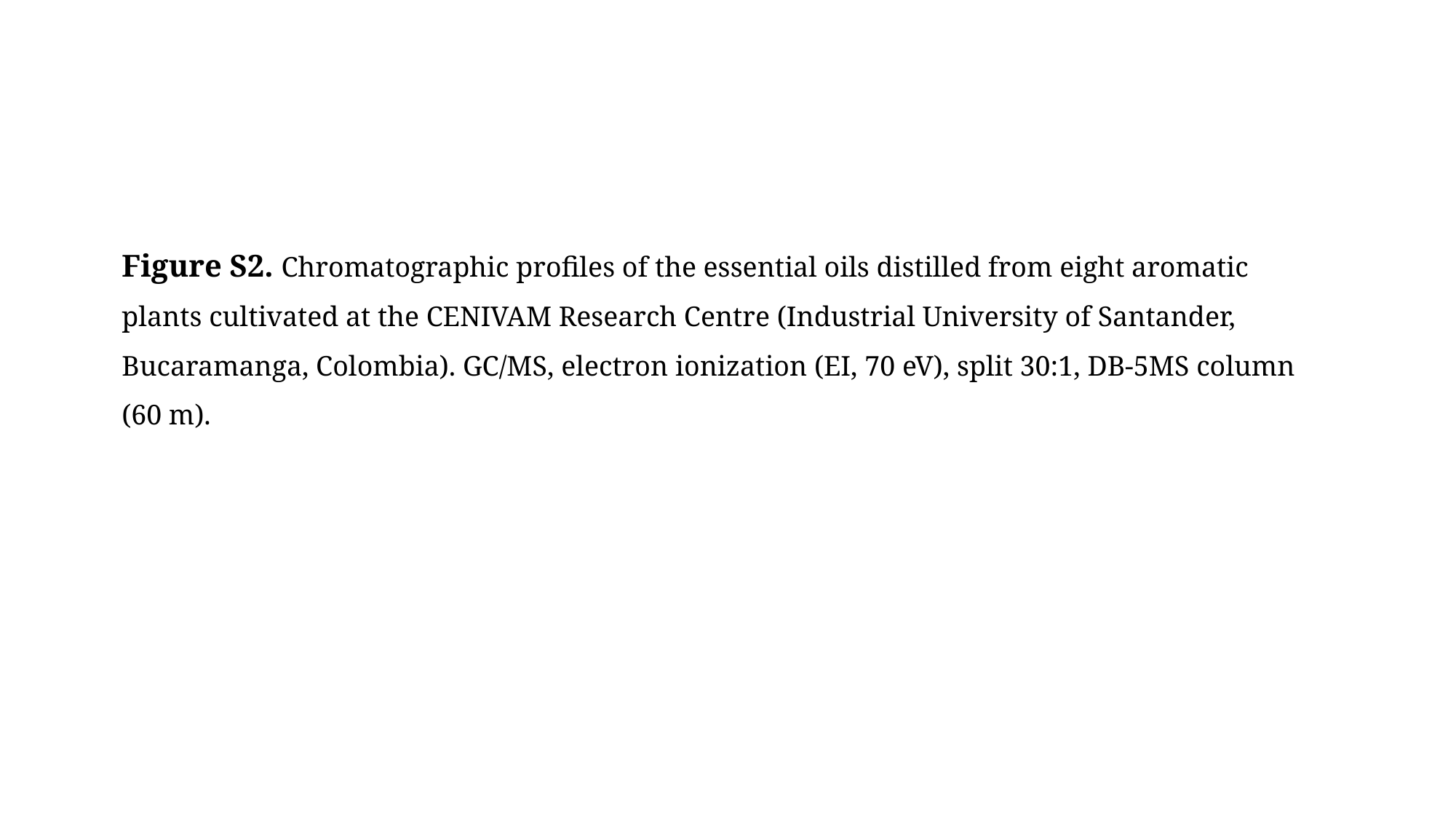

Figure S2. Chromatographic profiles of the essential oils distilled from eight aromatic plants cultivated at the CENIVAM Research Centre (Industrial University of Santander, Bucaramanga, Colombia). GC/MS, electron ionization (EI, 70 eV), split 30:1, DB-5MS column (60 m).

## Slide 6
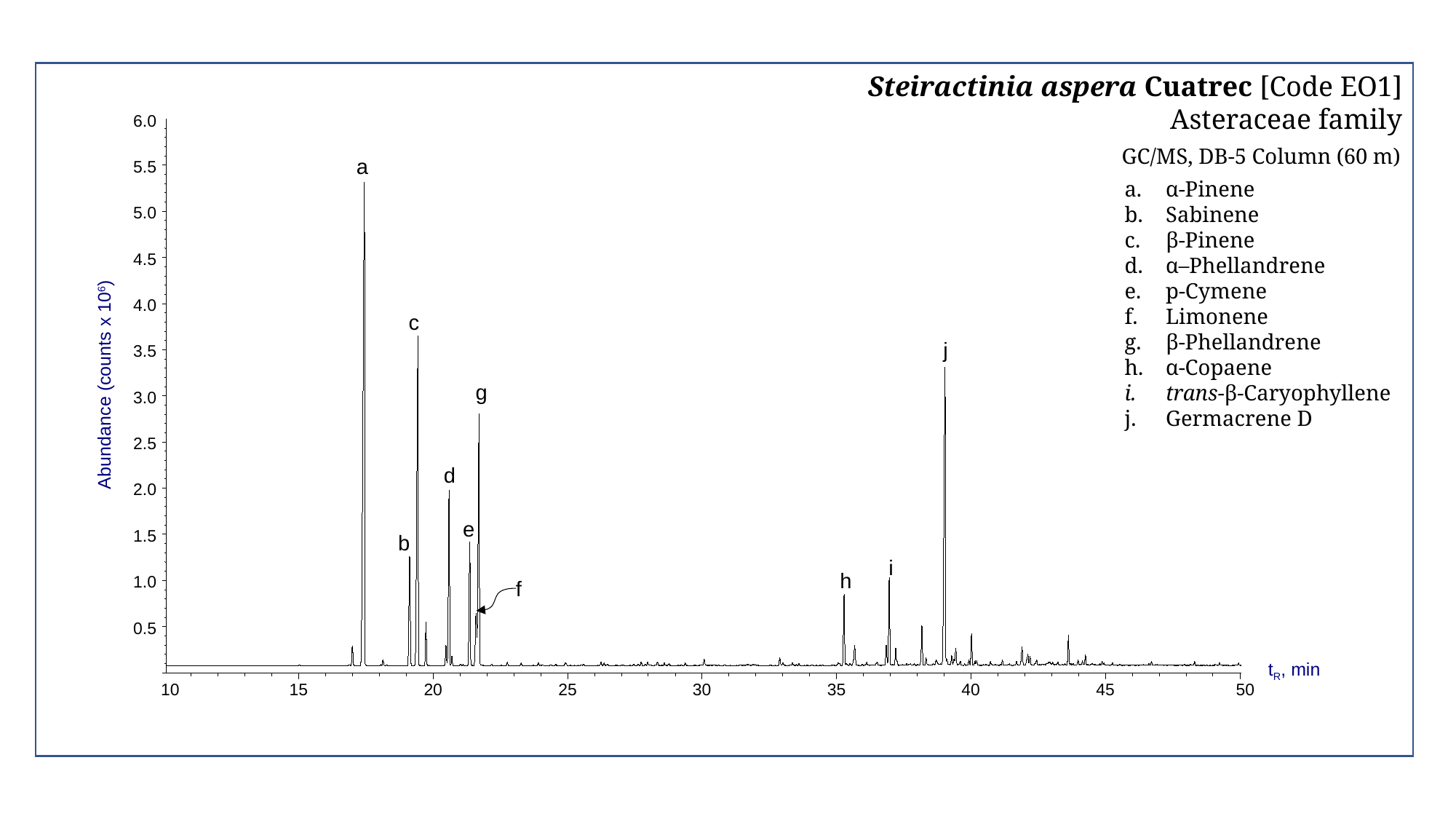

Steiractinia aspera Cuatrec [Code EO1]
Asteraceae family
6.0
a
5.5
α-Pinene
Sabinene
β-Pinene
α–Phellandrene
p-Cymene
Limonene
β-Phellandrene
α-Copaene
trans-β-Caryophyllene
Germacrene D
5.0
4.5
4.0
c
j
3.5
g
Abundance (counts x 106)
3.0
2.5
d
2.0
e
b
1.5
i
h
f
1.0
0.5
tR, min
10
15
20
25
30
35
40
45
50
GC/MS, DB-5 Column (60 m)

## Slide 7
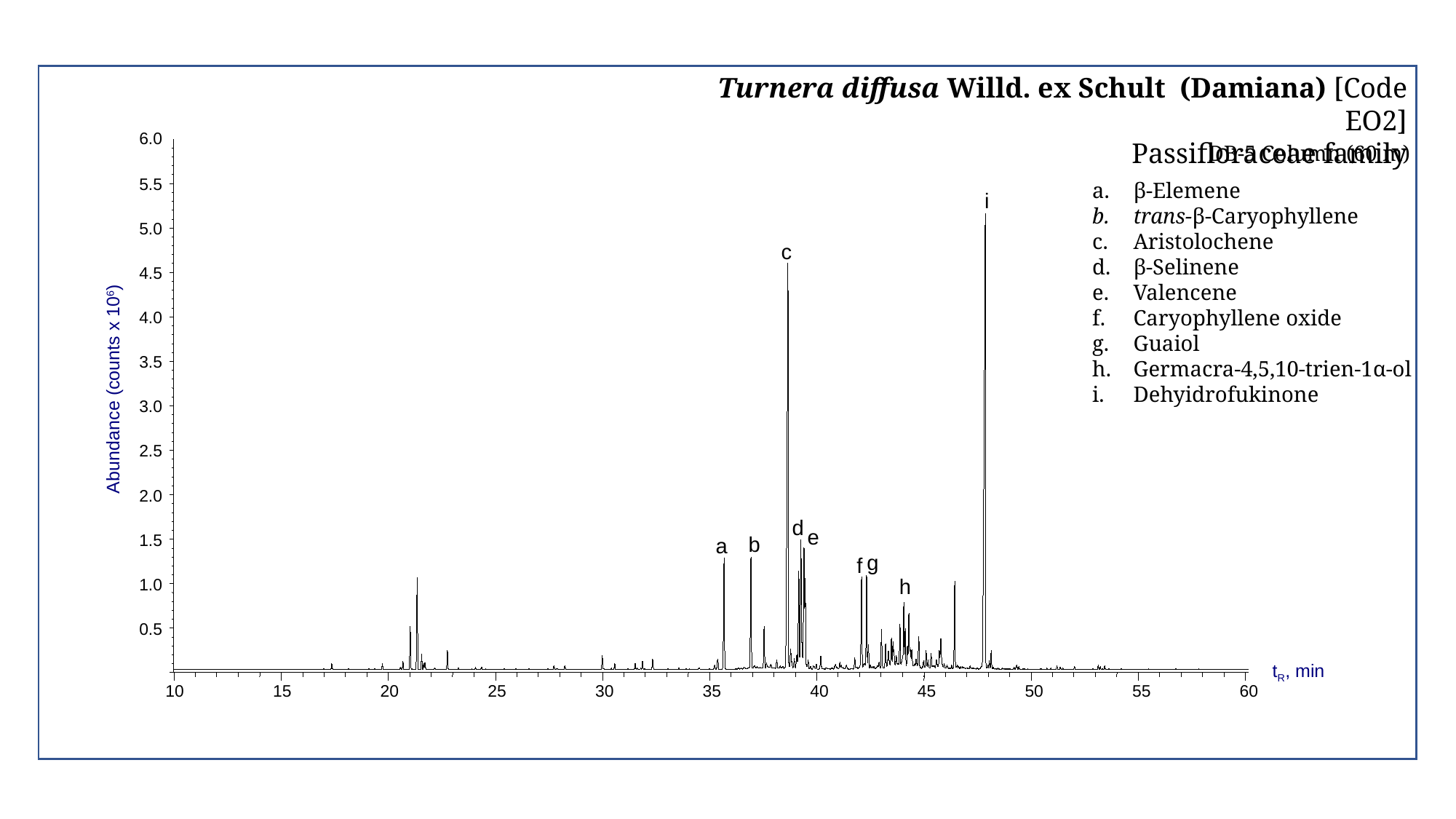

Turnera diffusa Willd. ex Schult (Damiana) [Code EO2]
Passifloraceae family
6.0
β-Elemene
trans-β-Caryophyllene
Aristolochene
β-Selinene
Valencene
Caryophyllene oxide
Guaiol
Germacra-4,5,10-trien-1α-ol
Dehyidrofukinone
5.5
i
5.0
c
4.5
4.0
3.5
Abundance (counts x 106)
3.0
2.5
2.0
d
e
b
a
1.5
g
f
h
1.0
0.5
tR, min
10
15
20
25
30
35
40
45
50
55
60
DB-5 Column (60 m)

## Slide 8
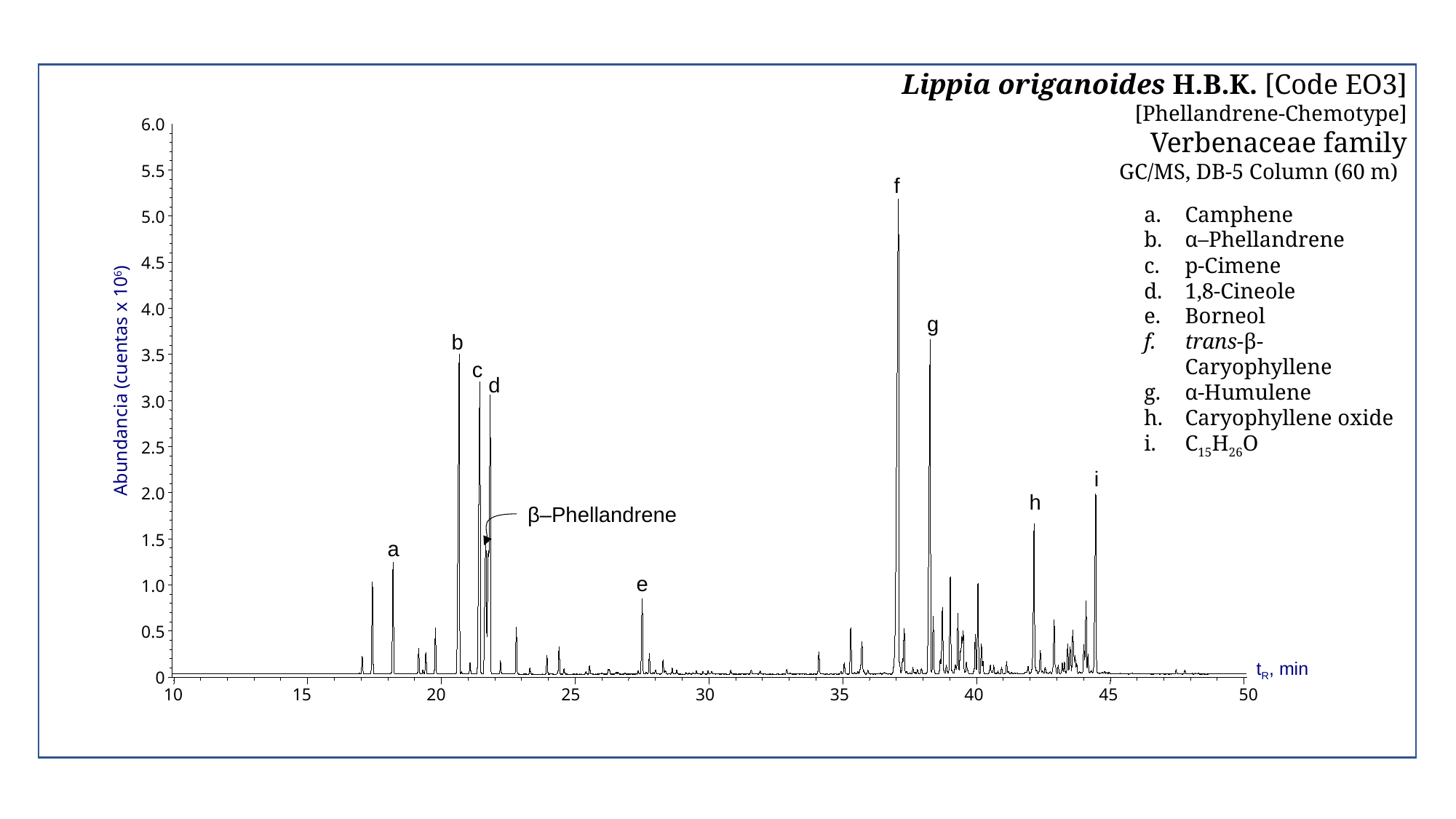

Lippia origanoides H.B.K. [Code EO3]
[Phellandrene-Chemotype]
Verbenaceae family
6.0
5.5
5.0
4.5
4.0
3.5
Abundancia (cuentas x 106)
3.0
2.5
2.0
1.5
1.0
0.5
0
10
15
20
25
30
35
40
45
50
GC/MS, DB-5 Column (60 m)
f
Camphene
α–Phellandrene
p-Cimene
1,8-Cineole
Borneol
trans-β-Caryophyllene
α-Humulene
Caryophyllene oxide
C15H26O
g
b
c
d
i
h
β–Phellandrene
a
e
tR, min

## Slide 9
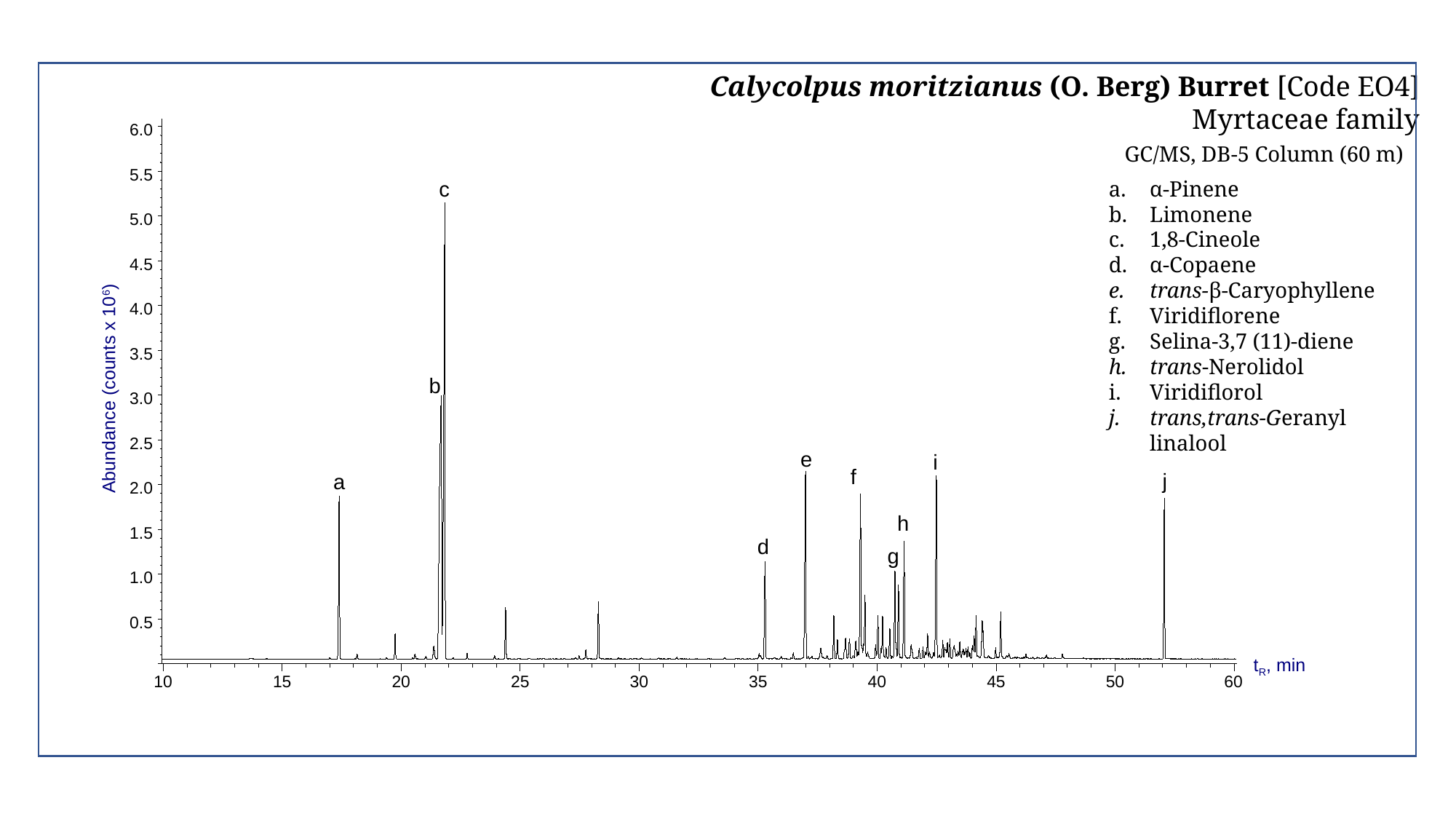

Calycolpus moritzianus (O. Berg) Burret [Code EO4]
Myrtaceae family
6.0
5.5
c
5.0
4.5
4.0
3.5
b
Abundance (counts x 106)
3.0
2.5
e
i
f
j
a
2.0
h
1.5
d
g
1.0
0.5
tR, min
10
15
20
25
30
35
40
45
50
60
GC/MS, DB-5 Column (60 m)
α-Pinene
Limonene
1,8-Cineole
α-Copaene
trans-β-Caryophyllene
Viridiflorene
Selina-3,7 (11)-diene
trans-Nerolidol
Viridiflorol
trans,trans-Geranyl linalool

## Slide 10
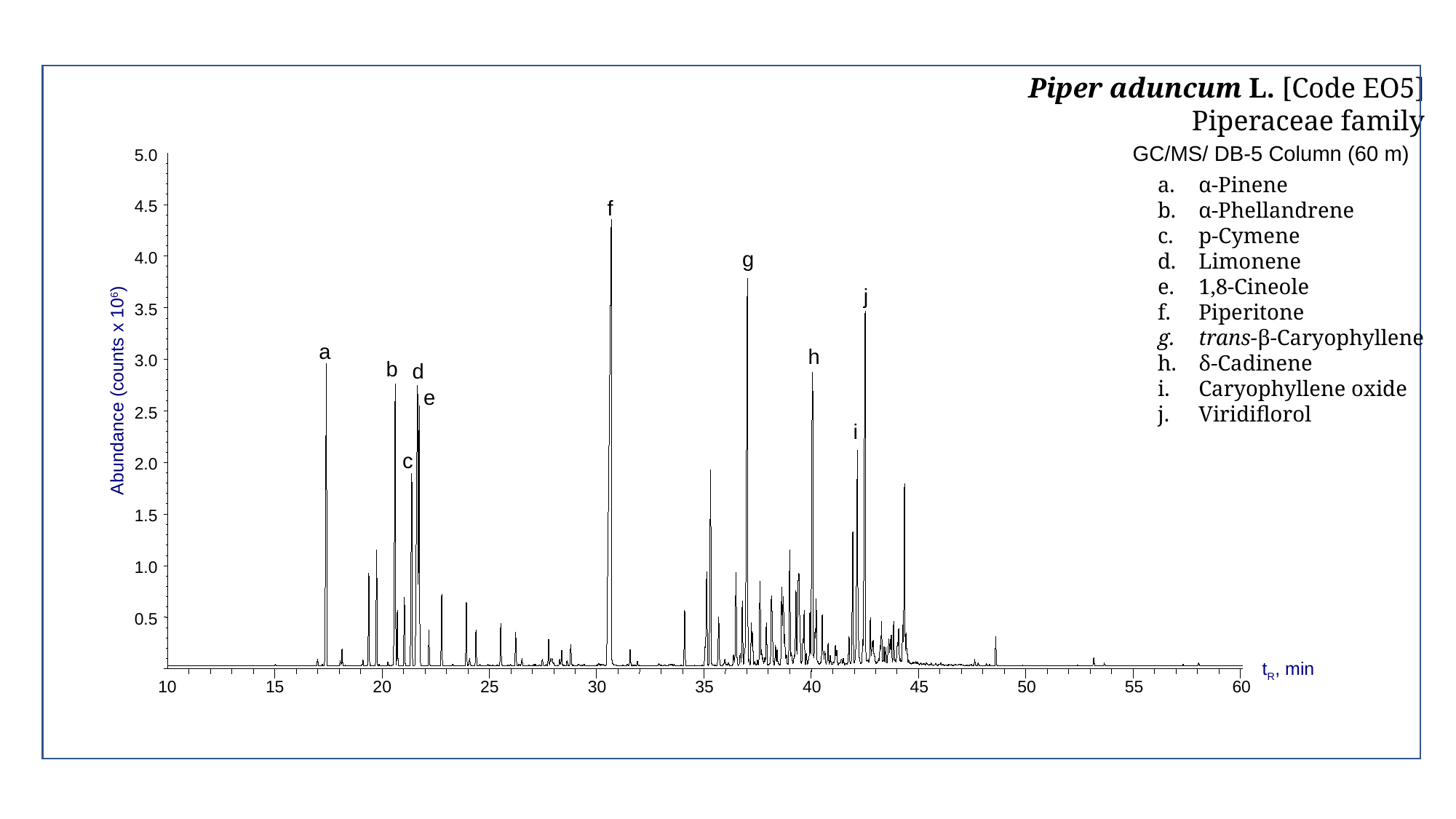

Piper aduncum L. [Code EO5]
Piperaceae family
GC/MS/ DB-5 Column (60 m)
5.0
f
4.5
g
4.0
j
3.5
a
h
b
3.0
d
e
Abundance (counts x 106)
2.5
i
c
2.0
1.5
1.0
0.5
tR, min
10
15
20
25
30
35
40
45
50
55
60
α-Pinene
α-Phellandrene
p-Cymene
Limonene
1,8-Cineole
Piperitone
trans-β-Caryophyllene
δ-Cadinene
Caryophyllene oxide
Viridiflorol

## Slide 11
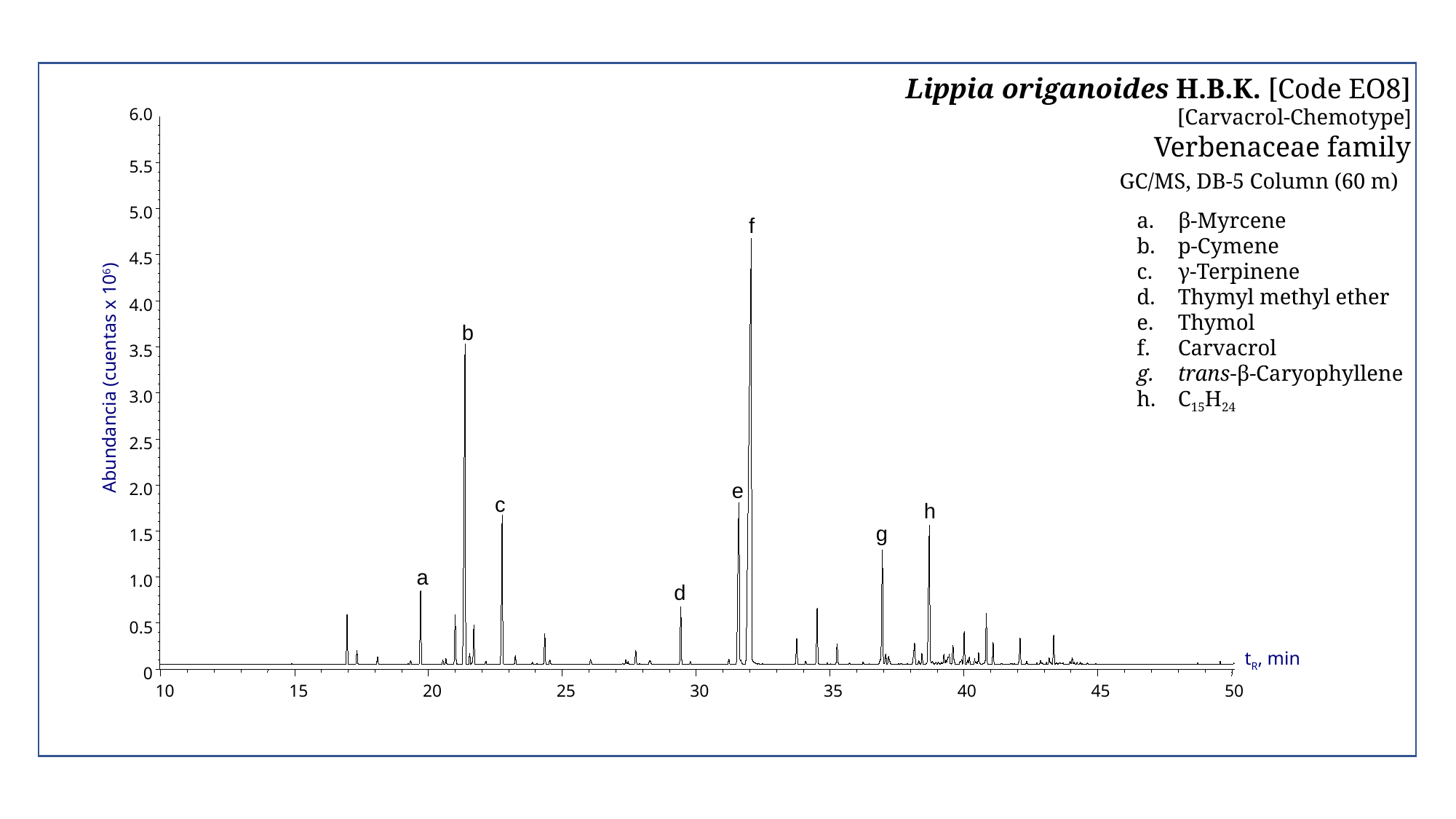

Lippia origanoides H.B.K. [Code EO8]
[Carvacrol-Chemotype]
Verbenaceae family
6.0
5.5
5.0
f
4.5
4.0
b
3.5
Abundancia (cuentas x 106)
3.0
2.5
e
2.0
c
h
g
1.5
a
1.0
d
0.5
tR, min
0
10
15
20
25
30
35
40
45
50
GC/MS, DB-5 Column (60 m)
β-Myrcene
p-Cymene
γ-Terpinene
Thymyl methyl ether
Thymol
Carvacrol
trans-β-Caryophyllene
C15H24

## Slide 12
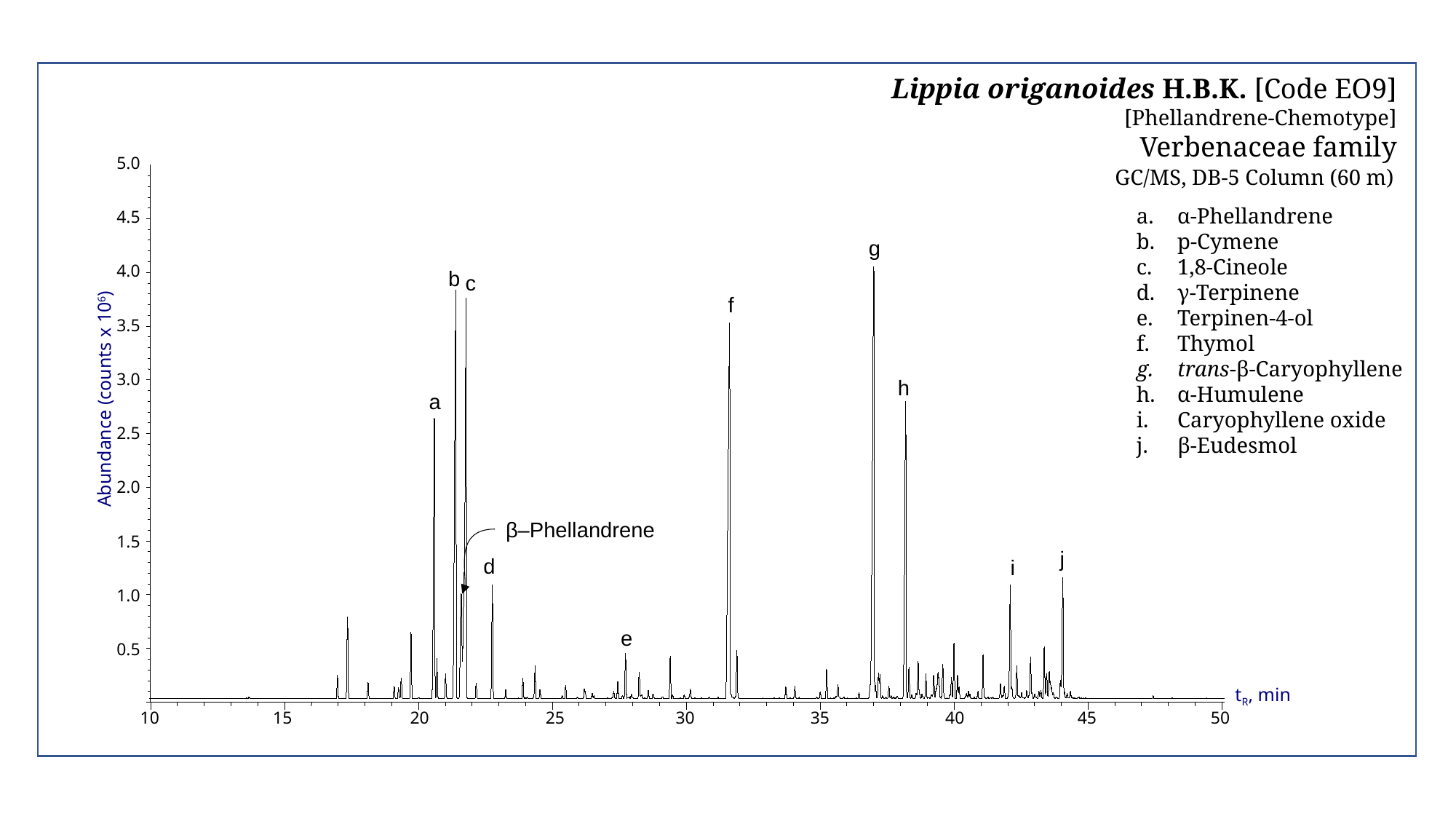

Lippia origanoides H.B.K. [Code EO9]
[Phellandrene-Chemotype]
Verbenaceae family
5.0
4.5
4.0
3.5
3.0
Abundance (counts x 106)
2.5
2.0
1.5
1.0
0.5
tR, min
10
15
20
25
30
35
40
45
50
GC/MS, DB-5 Column (60 m)
α-Phellandrene
p-Cymene
1,8-Cineole
γ-Terpinene
Terpinen-4-ol
Thymol
trans-β-Caryophyllene
α-Humulene
Caryophyllene oxide
β-Eudesmol
g
b
c
f
h
a
β–Phellandrene
j
d
i
e

## Slide 13
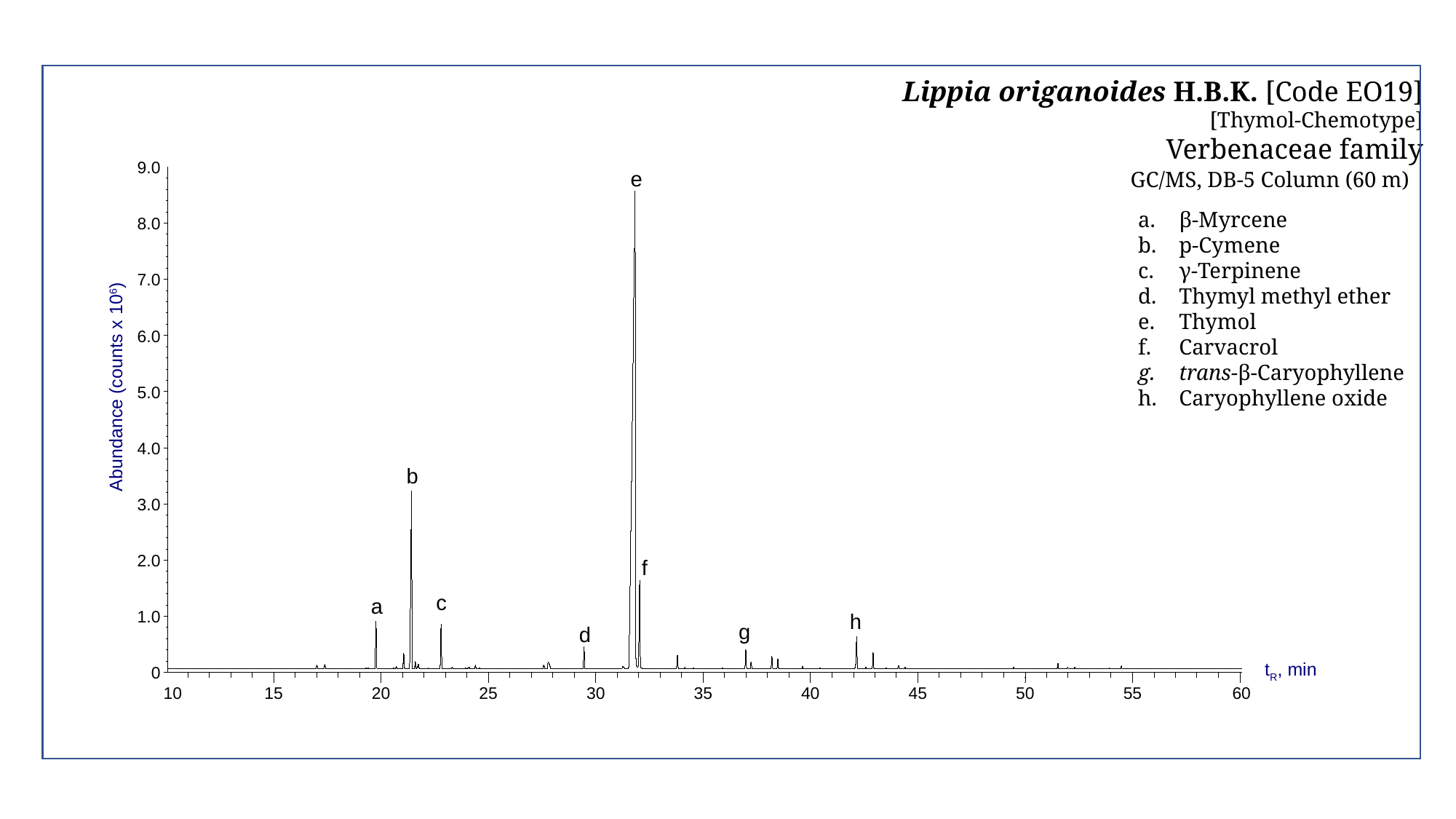

Lippia origanoides H.B.K. [Code EO19]
[Thymol-Chemotype]
Verbenaceae family
9.0
e
8.0
7.0
6.0
Abundance (counts x 106)
5.0
4.0
b
3.0
f
2.0
c
a
h
1.0
g
d
tR, min
0
10
15
20
25
30
35
40
45
50
55
60
GC/MS, DB-5 Column (60 m)
β-Myrcene
p-Cymene
γ-Terpinene
Thymyl methyl ether
Thymol
Carvacrol
trans-β-Caryophyllene
Caryophyllene oxide
