# Supplementary material for: Essential Oils Distilled from Colombian Aromatic Plants and Their Constituents as Penetration Enhancers for Transdermal Drug Delivery
Source: Molecules. 2023 Mar 22;28(6):2872. doi: 10.3390/molecules28062872 (PMC10057770; doi:10.3390/molecules28062872)
Supplement: Supplementary file 1 [file molecules-28-02872-s001.zip › Table S1_Supplementary_Material_P_Escobar (1).zip!/Table S1_Supplementary_Material_P_Escobar (1).pdf]

Table S1. Relative amounts (%) and linear retention indices (LPIs) of the principal compounds present in the essential oils studied.

Compound	LRIs				Relative GC peak áreas, %							
	Polar column**		Nonpolar column***									
	Exp.	Lit.[a]	Exp.	Lit.[a]	EO1	EO2	EO3	EO4	EO5	EO8	EO9	EO19
α-Pinene*	937	921–944	1021	1008–1039	24.9	-	2.0	5.1	4.6	0.4	1.7	0.2
Camphene*	952	936–959	1066	1043–1086	0.2	-	2.5	-	-	0.2	-	-
Sabinene*	976	961–981	1119	1098–1140	4.6	-	0.5	-	-	-	-	-
β-Pinene	982	964–988	1108	1085–1130	14.8	-	0.4	-	1.2	-	-	-
β-Myrcene*	990	980–995	1160	1140–1175	1.3	0.2	0.9	1.3	1.5	2.4	1.3	2.1
α-Phellandrene*	1009	995–1013	1163	1148–1186	6.3	-	9.3	-	4.4	-	7.1	-
Δ^3-Carene	1011	1002–1025	1146	1122–1169	0.3	0.2	-	-	0.6	0.1	0.7	-
α-Terpinene*	1019	1007–1026	1177	1154–1195	-	1.3	-	-	0.8	1.7	0.5	0.7
p-Cymene*	1028	1011–1033	1267	1246–1291	4.5	3.0	8.7	0.5	3.0	14.4	12.6	10.5
Limonene*	1033	1019–1039	1197	1178–1219	2.4	-	4.4	17.6	6.0	0.3	2.1	0.3
β-Phellandrene	1036	1011–1043	1206	1188–1233	10.1	-	3.1	-	-	-	1.4	-
1,8-Cineole*	1037	1021–1044	1212	1186–1231	-	-	6.5	19.1	3.6	1.3	13.0	0.3
γ-Terpinene*	1061	1049–1069	1244	1222–1266	-	0.6	1.0	-	0.8	5.3	2.4	2.0
Linalool*	1099	1088–1109	1545	1507–1564	-	-	0.5	1.3	0.4	1.0	0.7	0.2
Terpinen-4-ol*	1186	1165–1189	1603	1564–1630	-	-	0.4	-	0.3	0.5	0.9	0.7
Thymyl methyl ether	1232	1229–1240	1588	1563–1607	-	-	-	-	-	1.9	0.8	0.9
Piperitone	1264	1245–1266	1738	1689–1748	-	-	-	-	14.8	-	-	-
Thymol*	1290	1272–1304	2166	2100–2205	-	-	-	-	-	8.0	14.0	71.7
Carvacrol *	1301	1291–1314	2193	2140–2246	-	-	-	-	-	35.0	0.9	4.4
α-Copaene	1386	1363–1391	1497	1462–1522	2.6	-	1.1	3.2	2.9	0.7	0.6	-
β-Elemene	1397	1374–1402	1593	1565–1608	-	4.0	1.0	-	0.7	-	-	-
trans-β-Caryophyllene*	1435	1405–1440	1603	1569–1632	3.1	4.0	18.6	6.3	7.4	4.4	15.1	1.3
α-Humulene*	1469	1435–1470	1675	1637–1689	1.5	-	10.1	1.2	1.5	1.1	8.1	0.6
γ-Muurolene	1482	1461–1487	1691	1655–1714	0.2	-	1.2	0.9	1.5	-	0.6	-
Aristolochene	1483	1487[b]	1687	-	-	17.9	-	-	-	-	-	-

Compound	LRIs				Relative GC peak áreas, %							
	Polar column**		Nonpolar column***									
	Exp.	Lit.[a]	Exp.	Lit.[a]	EO1	EO2	EO3	EO4	EO5	EO8	EO9	EO19
Germacrene D*	1494	1464–1493	1710	1676–1726	13.1	-	2.2	-	1.7	-	0.9	-
Premnaspirodiene	1498	1505[b]	1725	-	-	3.7	-	-	-	-	-	-
β-Selinene	1501	1473–1496	1723	1686–1743	-	5.2	1.2	2.5	-	0.3	0.5	-
Valencene	1505	1477–1507	1713	1688–1761	-	7.4	-	0.3	1.2	-	-	-
α-Selinene	1509	1477–1510	1726	1696–1748	-	1.6	0.9	1.7	-	0.8	-	-
γ-Cadinene	1524	1498–1531	1760	1735–1782	0.2	-	0.9	0.4	0.7	0.3	0.5	-
δ-Cadinene	1527	1508–1539	1755	1722–1774	1.0	-	2.0	1.2	5.5	1.1	1.2	-
Selina-3,7(11)-diene	1551	1531–1546	1780	1750–1800	-	-	-	2.8	-	-	-	-
trans-Nerolidol*	1563	1539–1570	2035	1995–2055	-	-	-	3.5	-	0.7	0.9	-
Caryophyllene oxide*	1597	1563–1595	1981	1936–2023	0.8	3.2	3.8	0.7	3.8	0.9	2.5	1.6
Guaiol*	1606	1585–1615	2084	2061–2104	-	3.5	0.5	-	-	-	0.6	-
Viridiflorol	1610	1569–1604	2097	2041–2110	0.3	-	-	5.7	6.5	-	-	-
β-Eudesmol	1670	1637–1664	2213	2196–2272	-	-	1.5	0.7	0.8	0.3	2.6	0.2
Germacra-4,5,10-trien-1-α-ol	1680	1686[b]	2365	-	-	3.5	-	-	-	-	-	-
Dehydrofukinone	1827	1820[c]	2448	2404[c]	-	25.4	-	-	-	-	-	-
trans,trans-Geranyl linalool	2028	2026[b]	-	2551[c]	-	-	-	4.0	-	-	-	-

Exp. – Experimental LRI; **Lit.** - Literature. * Confirmatory identification based on the use of standard compounds. ** DB-WAX Column (60 m). *** DB-5MS Column (60 m).

[a] [90% LRI range] V. I. Babushok, P. J. Linstrom and I. G. Zenkevich (2011) Retention Indices for Frequently Reported Compounds of Plant Essential Oils, J. Phys. Chem. Ref. Data, 40, 043101. https://doi.org/10.1063/1.3653552.

[b] R. P. Adams (2007) Identification of essential oil components by gas chromatography/mass spectrometry. 4th Ed., Allured Publisher, Carol Stream, IL, U.S.A., pp. 1-809. https://doi.org/10.1016/j.jasms.2005.07.008.

[c] W. Wallace (2017) NIST Standard Reference Database 1A. Version 2.3.
